# Surfactant-Assisted Hydrothermal Synthesis of PMN-PT Nanorods

**DOI:** 10.1186/s11671-016-1253-8

**Published:** 2016-02-01

**Authors:** Chuan Li, Xingzhao Liu, Wenbo Luo, Dong Xu, Kai He

**Affiliations:** State Key Laboratory of Electronic Thin Film and Integrated Devices, School of Microelectronics and Solid-State Electronics, University of Electronic Science and Technology of China, Chengdu, 610054 China; School of Material Science and Engineering, Jiangsu University, Zhenjiang, 212013 China

**Keywords:** 0.7PMN-0.3PT, Nanorods, Piezoelectric materials, Hydrothermal, Microstructure

## Abstract

The effects of surfactant polyacrylate acid (PAA) on shape evolution of 0.7Pb(Mg_1/3_Nb_2/3_)O_3_-0.3PbTiO_3_ (0.7PMN-0.3PT) nanorods were studied. The results revealed that the polyacrylic acid content had great influence on the morphology of 0.7PMN-0.3PT. With increasing PAA concentration from 0.45 to 0.82 g/ml, the ratio of perovskite phase (PMN-PT nanorod) increased, while the ratio of pyrochlore phase decreased. When the PAA concentration was 0.82 g/ml, pure 0.7PMN-0.3PT nanorods were obtained. However, when PAA concentration was higher than 0.82 g/ml, the excess of PAA would hindered their [100] orientation growth. The piezoelectric coefficient *d*_33_ of 0.7PMN-0.3PT nanorod was obtained by linear fitting, and the *d*_33_ value was 409 pm/V.

## Background

Perovskite-typed ferroelectric materials have relatively high piezoelectric constant, such as (1-*x*)PbZn_1/3_Nb_2/3_O_3_-*x*PbTiO_3_ (PZNT) [1], Pb-based lanthanum-doped zirconate titanates (PZT) [2], and BaTiO_3_ [3], which have been widely applied in the self-powered sensors [4–6] and energy conversion devices [7, 8]. Some non-ferroelectric lead-free materials also have good piezoelectric properties, such as ZnO [9] nanorod-based nanogenerator could provide an output voltage of 2 V, which has been demonstrated to drive a small light-emitting diode (LED) [10]. However, the actual publication may need more powerful piezoelectric material to make nanodevices.

Compared with other piezoelectric materials, a solid solution (1*-x*)Pb(Mg_1/3_Nb_2/3_)O_3_-*x*PbTiO_3_ (PMN-PT) compound is promising for both further research and applications due to its high piezoelectric effect of 2500 pm/V [11–13]. Sun et al. [14] indicated that the nanorod of PMN-PT are good candidates than bulks in mechanical energy harvesting due to their high strain tolerance and the larger output power generated by PMN-PT. The piezoelectric properties of the PMN-PT ceramics may have been studied, but the PMN-PT nanorod rarely has been studied systematically. Xu et al. [15] firstly synthesized the 0.72PMN-0.28PT nanorod via hydrothermal method; it has a piezoelectric constant 15 times higher than ZnO nanowire and 3 times higher than PZT nanowire.

Hydrothermal method is a versatile method to synthesize ceramic nanoparticles and nanowires. The hydrothermal reaction is a complicated progress; a tiny vary in the synthesis progress would change the final morphology of the product. In this letter, we investigated the influences of the surface active agent on the synthesis progress of the PMN-PT nanorods. It is found that the polyacrylic acid content could have great influence on morphology of the PMN-PT nanorods. The piezoelectric coefficient of 0.7PMN-0.3PT nanorod has also been tested.

## Methods

### Synthesis the PMN-PT Nanorods

The hydrothermal method was used to synthesis the PMN-PT nanorods. Poly(ethylene glycol)-200 (PEG200) and methanol (MeOH) mixture (PEG200/MeOH at a 1:2 volume ratio) were used as the solvent for the starting materials, and 1,1,1-tris (methylol)ethane (THOME) was used as the complexing agent. Stoichiometric amounts (corresponding to the 0.7PMN-0.3PT composition) of lead acetate trihydrate, magnesium 2,4-pentanedionante dehydrate (MgAA), niobium ethoxide, and titanium diisopropoxide bisacetyl acetonate (TIAA) were used to prepare the Pb-Mg-Nb-Ti (PMNT) sol-gel. Lead acetate trihydrate, MgAA, and THOME were mixed with PEG200/MeOH mixture with strong stirring to form a Pb-Mg solution. In a separate reaction flask, niobium ethoxide, TIAA, and THOME were also mixed with PEG200/MeOH mixture with strong stirring to form an Nb-Ti solution. After stirring for 2 h at room temperature, these two solutions were then mixed together and stirred for about 4 h to form a clear yellow PMNT sol. The PMNT sol was dispersed into deionized (DI) water with strong stirring in the ratio of 1:10. Polyacrylic acid was introduced to the solution as surfactants to produce PMN-PT nanorods. Then, KOH was added into the yellow solution with quickly stirring and a white precipitate was formed. The suspension was sealed into a Teflon-lined stainless steel autoclave with the capacity of 80 ml and kept in an oven at 235 °C for 24 h. After cooling to room temperature, the suspension was washed with ethanol and DI water six times and dried at 100 °C in an oven for 6 h. Grey powder consisted of 0.7PMN-0.3PT nanorods was obtained.

### Measurements

The morphology characteristics of products were determined by scanning electron microscope (SEM, Inspect F50). The structure and phase composition were investigated by powder X-ray diffractometer (XRD, DX1000) with Cu Kα of 0.1542 nm (40 kV, 30 mA) radiation. The microstructure of the PMN-PT nanorod was investigated by transmission electron microscopy (TEM, Tecnai G2 F20 S-TWIN). The piezoelectric coefficient of single PMN-PT nanorods was measured by atomic force microscopy (AFM, SEIKO SPA300HV) using a contact model.

## Results and Discussion

**Fig. 1:**
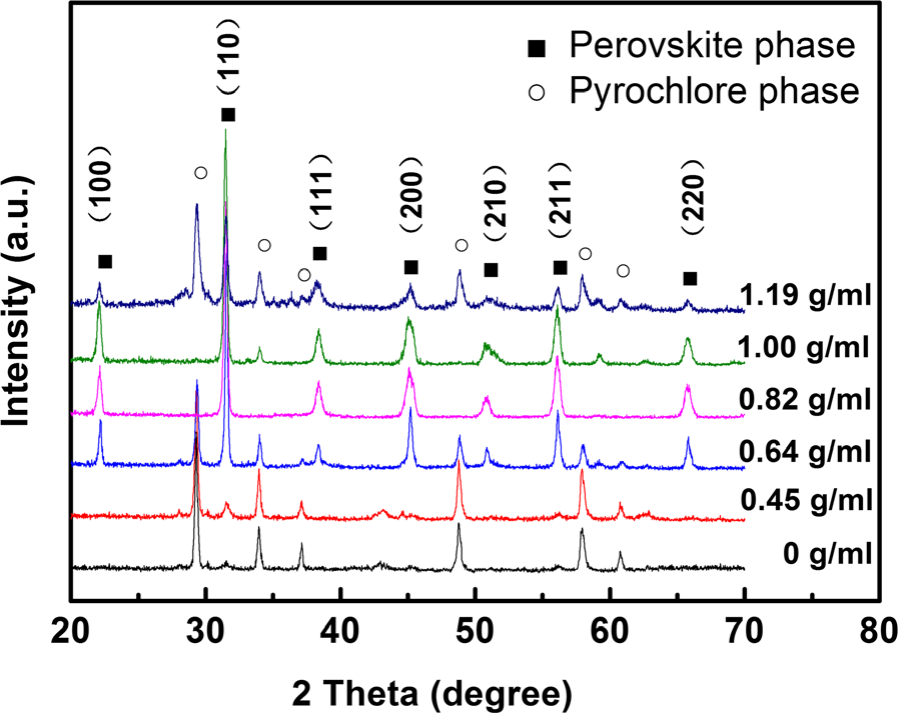
XRD pattern of PMN-PT synthesized at 235 °C with different concentration of PAA

To study the effects of different polyacrylate acid (PAA) contents on the formation of PMN-PT nanorods, the hydrothermal reaction was carried out at 235 °C for 24 h. The concentrations of PAA are 0, 0.45, 0.64, 0.82, 1.0, and 1.19 g/ml, respectively. The phases of corresponding samples were checked by XRD with Cu Kα of 0.1542 nm radiation. The XRD pattern of samples synthesized with different PAA concentrations are shown in Fig. 1. It could be indicated that the pyrochlore phases appear as the major phase with PAA concentration of 0 and 0.45 g/ml. The diffraction peaks of perovskite phase appeared, and its intensity is enhanced with the increasing of PAA concentration. It means the perovskite phase grows at the expense of the pyrochlore phase. Once the hydrothermal condition satisfied the threshold of nucleation, PMN-PT nuclei would form at the initial stage. The functional groups in PAA would tend to absorb on the surface of PMN-PT nuclei resulting in the surface energy decrease. The perovskite nuclei would be thermodynamically stable and could start to grow. An almost pure perovskite phase appears at PAA concentration of 0.82 g/ml. The diffraction peaks reveal that PMN-PT nanorods have good crystallized. However, the pyrochlore phase appears as the concentration of PAA increases above 0.82 g/ml. The amount of pyrochlore phase increases as PAA concentration increased. At high PAA concentration, the PAA attached to the PMN-PT nuclei surface in the form of a shell at the nucleation stage. The polymer shell will inhibit the crystal growth of PMN-PT nuclei.

**Fig. 2:**
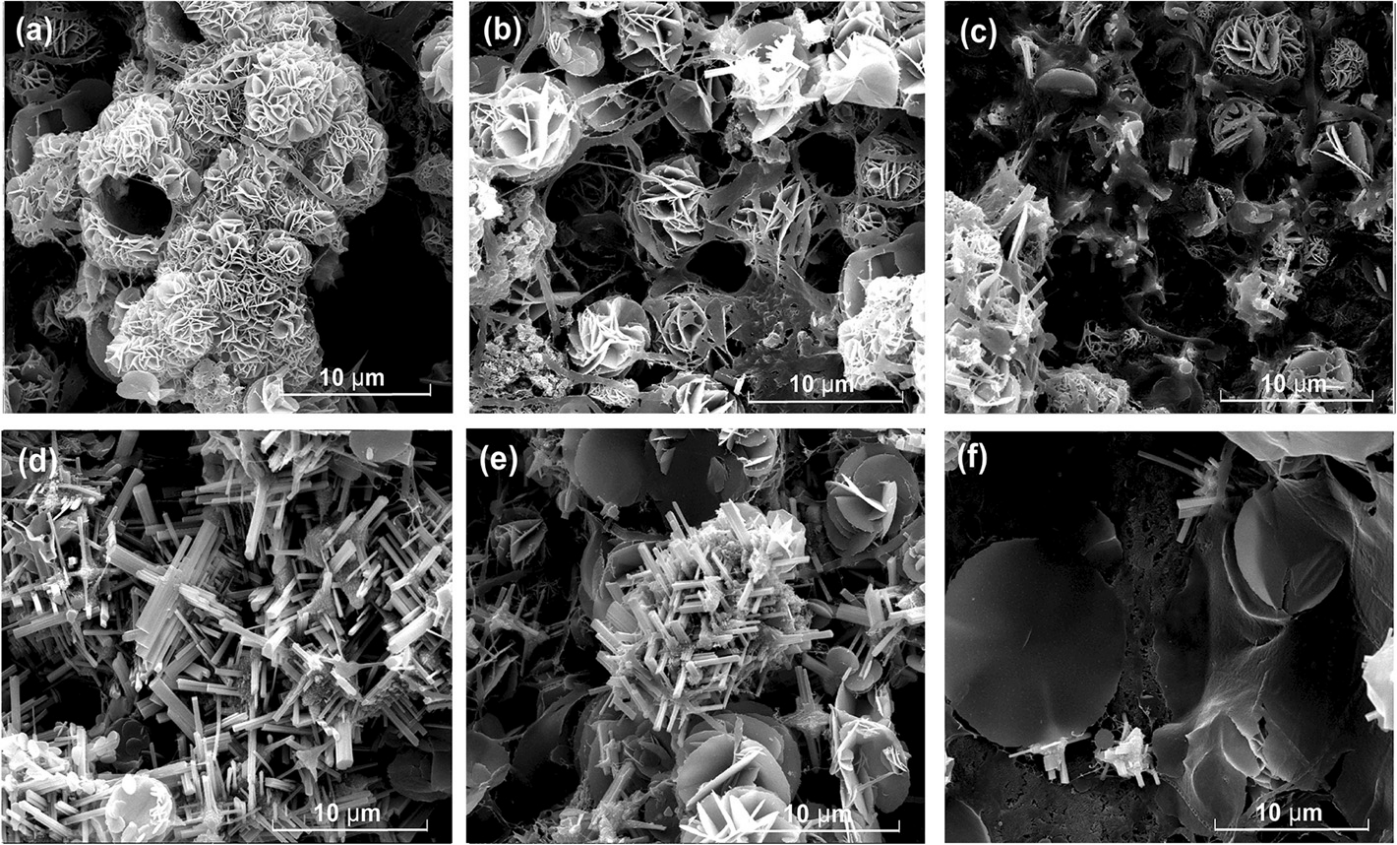
SEM images of the products with PAA concentration of 0 g/ml (**a**), 0.45 g/ml (**b**), 0.64 g/ml (**c**), 0.82 g/ml (**d**), 1.0 g/ml (**e**), and 1.19 g/ml (**f**)

The morphology of samples synthesized with different concentrations of PAA are shown in Fig. 2. There is almost no nanorod product but cluster of nanoflowers when the concentration of PAA is 0 and 0.45 g/ml. According to XRD results, the morphology of the pyrochlore phase is nanoflower-like. As the concentration of PAA increases from 0.45 to 0.82 g/ml, more nanorods formed and the ratio of nanoflower decreased. As we know, it needs more energy to form perovskite structure than pyrochlore. The functional groups of PAA would improve the adsorption behaviors on the PMN-PT nuclei surface. The surface energy of different crystal faces has strong influence on the growth speed. At low PAA concentration, not all the crystal growth surface contacted with PAA. The added polymers perform as capping agents that chemisorb at the side surfaces of the nanorod during the growth of PMN-PT nuclei [16]. Therefore, the length of the PMN-PT nanorod increases along [100] orientation, and the growth in the radial direction is hindered. Pure nanorod products with perovskite structure are obtained when the concentration of PAA is 0.85 g/ml. However, the amount of nanorods decreases and the length of the rod got shorter when the concentration of PAA is increasing from 0.82 to 1.19 g/ml. It could be due to the excess of PAA adsorbed on the surface of PMN-PT nuclei as a shell which hindered the crystal growth of PMN-PT nuclei at the nucleation stage. Meanwhile, the morphology of pyrochlore phase becomes nanoflake.

**Fig. 3:**
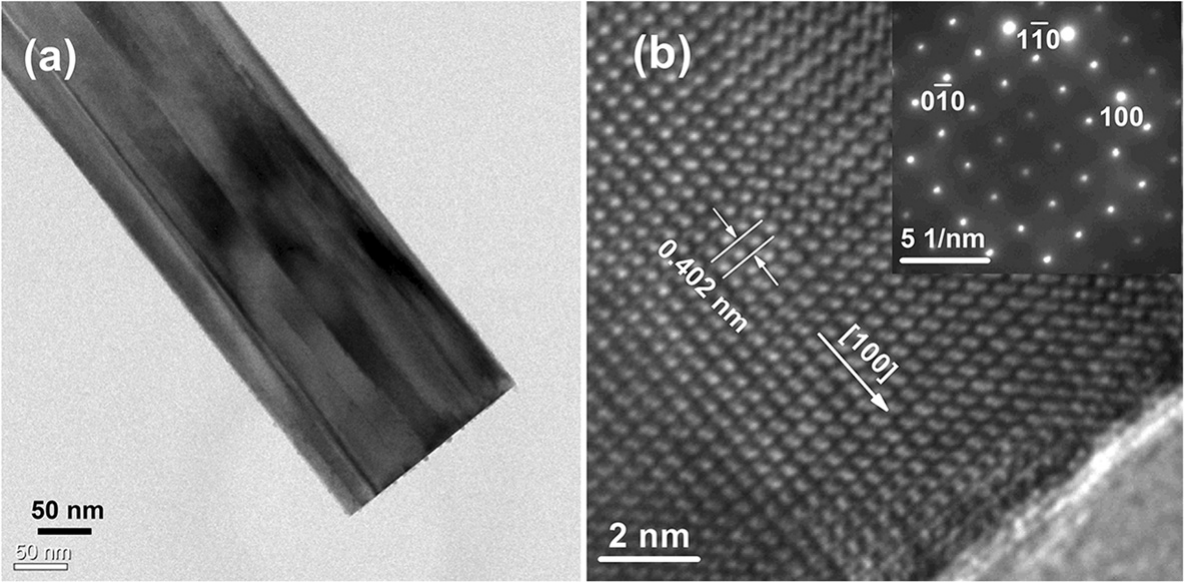
**a** Low-magnification TEM image of PMN-PT nanorod. **b** High-angle annular dark-field (HAADF) image of a nanorod and the insert is electron diffraction pattern

The morphologies, microstructures, and crystalline phases of the PMN-PT nanorods were performed by TEM and SAED (selected area electron diffraction), as shown in Fig. 3. Low-magnification TEM image of Fig. 3a shows that the nanorods have cuboid morphology and the size in radial direction is about 193 nm. The high-resolution TEM image shown in Fig. 3b further reveals that the nanorods have a good single crystal structure. Moreover, the lattice spacing of 0.402 nm corresponds to the (100) lattice plane, indicating the nanorod grow along [100] direction. The inset SAED pattern in Fig. 3b reveals that PMN-PT nanorods are structurally uniform and single crystal.

**Fig. 4:**
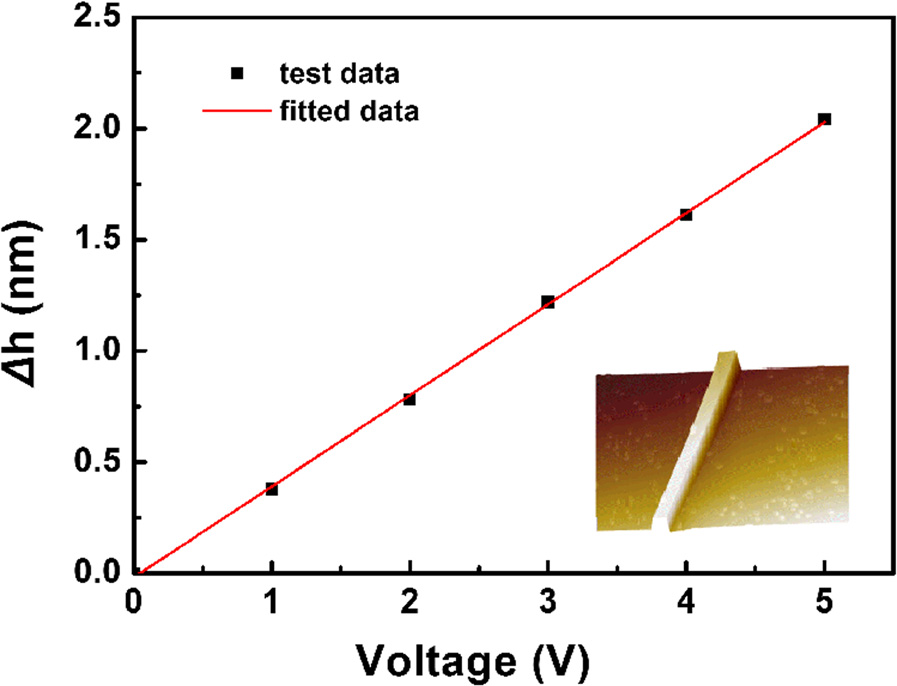
Piezoelectric displacement vs voltage curve of PMN-PT nanorod, with 3D morphology inserted

The PMN-PT nanorods were dispersed by ethanol. Then, a drop of suspension was put on Au/Ti-covered silicon wafer and dried in air. The *d*_33_ coefficient of single PMN-PT nanowire was measured by AFM using a contact model. The relationship of piezoelectric displacement and voltage applied on AFM tip was plotted in Fig. 4, with 3D AFM image inserted. The equation for the fitted curve (red line) is displacement = −0.01895 + 0.409 × voltage, which means the piezoelectric coefficient *d*_33_ determined from the slope of the linear fitting is 409 pm/V. This value is slightly larger than *d*_33_ value of 0.72PMN-0.28PT nanorod (381 pm/V) [15]. It is worth noting that the *d*_33_ value of PMN-PT nanorod is much lower than the PMN-PT ceramics. This is due to the fact that the PMN-PT nanorod is not been polarized. The ferroelectric domains of the nanorod, as we can see in Fig. 3a, have different spontaneous polarization directions. Moreover, the *d*_33_ value of PMN-PT nanorod is measured along [010] or [001] direction, but the *d*_33_ value of textured PMN-PT ceramics is measured along [111] direction.

## Conclusions

Single crystalline PMN-PT nanorods of cuboid morphology were obtained at 235 °C with the addition of PAA in hydrothermal reaction. As PAA concentration increased from 0.45 to 0.82 g/ml, the length of the PMN-PT nanorod increased while the growth in the radial direction was hindered. An almost pure perovskite phase (PMN-PT nanorod) appeared at a PAA concentration of 0.82 g/ml. The orientation growth of PMN-PT was attributed to the surface energy difference caused by absorption of PAA on different crystal plane. The piezoelectric coefficient of 0.7PMN-0.3PT nanorod was 409 pm/V, which provides a great potential application in manufacturing piezoelectric nanodevices.

## Abbreviations

AFM: atomic force microscopy
DI: deionized
HAADF: high-angle annular dark-field
LED: light-emitting diode
MeOH: methanol
MgAA: magnesium 2,4-pentanedionante dehydrate
PAA: polyacrylate acid
PEG200: poly(ethylene glycol)-200
PMN-PT: (1*-x*)Pb(Mg_1/3_Nb_2/3_)O_3_-*x*PbTiO_3_
PMNT: Pb-Mg-Nb-Ti
PZNT: (1-*x*)PbZn_1/3_Nb_2/3_O_3_-*x*PbTiO_3_
PZT: Pb-based lanthanum-doped zirconate titanates
SAED: selected area electron diffraction
SEM: scanning electron microscope
TEM: transmission electron microscopy
THOME: 1,1,1-tris (methylol)ethane
TIAA: titanium diisopropoxide bisacetyl acetonate
XRD: X-ray diffractometer
